# Prognostic Role of Worst Pattern of Invasion in Oral Squamous Cell Carcinoma

**DOI:** 10.3390/jcm15030965

**Published:** 2026-01-25

**Authors:** Lucrezia Togni, Marco Mascitti, Paolo Maria Antonio Staffinati, Giuseppe Consorti, Gaetano Isola, Lucio Lo Russo, Andrea Santarelli

**Affiliations:** 1 Department of Clinical Specialistic and Dental Sciences, Marche Polytechnic University, Via Tronto 10/A, 60126 Ancona, Italy; l.togni@staff.univpm.it (L.T.); s1131815@pm.univpm.it (P.M.A.S.); giuseppe.consorti@ospedaliriuniti.marche.it (G.C.); andrea.santarelli@staff.univpm.it (A.S.); 2 Division of Maxillofacial Surgery, Department of Neurological Sciences, Marche University Hospitals-Umberto I, Via Conca 71, 60126 Ancona, Italy; 3 Department of General Surgery and Surgical-Medical Specialties, School of Dentistry, University of Catania, Via S. Sofia 78, 95124 Catania, Italy; gaetano.isola@unict.it; 4 Department of Clinical and Experimental Medicine, University of Foggia, Via Rovelli 50, 71122 Foggia, Italy; lucio.lorusso@unifg.it; 5 Dentistry Clinic, National Institute of Health and Science of Aging, IRCCS INRCA, Via Tronto 10/A, 60126 Ancona, Italy

**Keywords:** oral squamous cell carcinoma, oral tongue squamous cell carcinoma, pattern of invasion, Worst Pattern of Invasion, tumor microenvironment, head and neck cancer, prognosis

## Abstract

**Background/Objectives**: The pattern of invasion describes the arrangement of neoplastic cells along the tumor infiltrative front and refers to the way cancer infiltrates tissue at the tumor/host interface. Accumulating evidence suggested that the Worst Pattern of Invasion (WPOI) represents an independent prognostic factor in oral squamous cell carcinoma (OSCC). However, it is still considered a minor prognostic criterion, and it is recommended as an optional report component in the College of American Pathologists (CAP) guideline. **Methods**: Therefore, the study aims to extensively review the literature data regarding the prognostic role of the WPOI in OSCC. **Results**: The WPOI resulted as an independent prognostic factor for locoregional recurrences (LRRs), lymph node metastasis (LMN), overall survival (OS), disease-specific survival (DSS), and bone tissue infiltration, regardless of the oral subsite and the pathological stage. Moreover, several authors suggested the evaluation of the WPOI to lead the postoperative management and to determine the occult LNM in early-stage OSCC. **Conclusions**: The prognostic relevance of the WPOI in OSCC highlights its evaluation in pathological daily practice. Therefore, the WPOI-detection method and scoring system should be validated based on the tumor stage and site.

## 1. Introduction

Oral squamous cell carcinoma (OSCC) represents over the 90% of all oral cavity cancers, with over 377.700 cases diagnosed annually worldwide. In 2025, the National Cancer Institute estimated about 59,660 new cases and 12,770 new deaths, with a short-term survival rate (2015–2021) equal to 69.5%, ranging from 88.4% (localized cancers) to 36.4% (advanced cancers) [1]. OSCC can develop from any oral region; however, the tongue is the most involved oral subsite, accounting for 41% of all cases [2]. Notably, oral tongue squamous cell carcinoma (OTSCC) seems to exhibit distinctive molecular and clinical behavior compared to oral cancer of other subsites, leading to an “anatomical bias” both for research and for clinical decision making [3,4].

Despite the advancements of the 8th edition of American Joint Committee on Cancer (AJCC) staging system, it exhibits suboptimal risk stratification and prognostication, and the survival outcomes of patients classified as having the same pathological stage are very different. The AJCC staging system is primarily based on clinicopathological features, without considering the complex biological heterogeneity of the tumor microenvironment (TME) [5,6], a complex ecosystem interposed between the malignant cells and normal host tissues [7]. To improve the OSCC stratification and accuracy, in the last decade, researchers have been strongly focusing on the evaluation of the infiltrative tumor front. It represents the area of active neoplastic invasion, and the crosstalk between cancer cells and the TME is considered an active promoter of cancer progression [8,9,10,11]. These interactions affect the ways in which cancer cells infiltrate adjacent tissues at the tumor–host interface, resulting in extreme variability of histological patterns [12]. Among the histological parameters developed and proposed to investigate this heterogeneity, the pattern of invasion (POI) has been increasingly recognized as a relevant parameter. It describes the arrangement of neoplastic cells along the tumor infiltrative front and refers to the way that cancer infiltrates the tissue at the tumor/host interface. Therefore, based on the morphological features of the infiltrative front, several classifications of the POI have been proposed [13,14,15,16,17,18,19,20,21,22].

**Figure 1:**
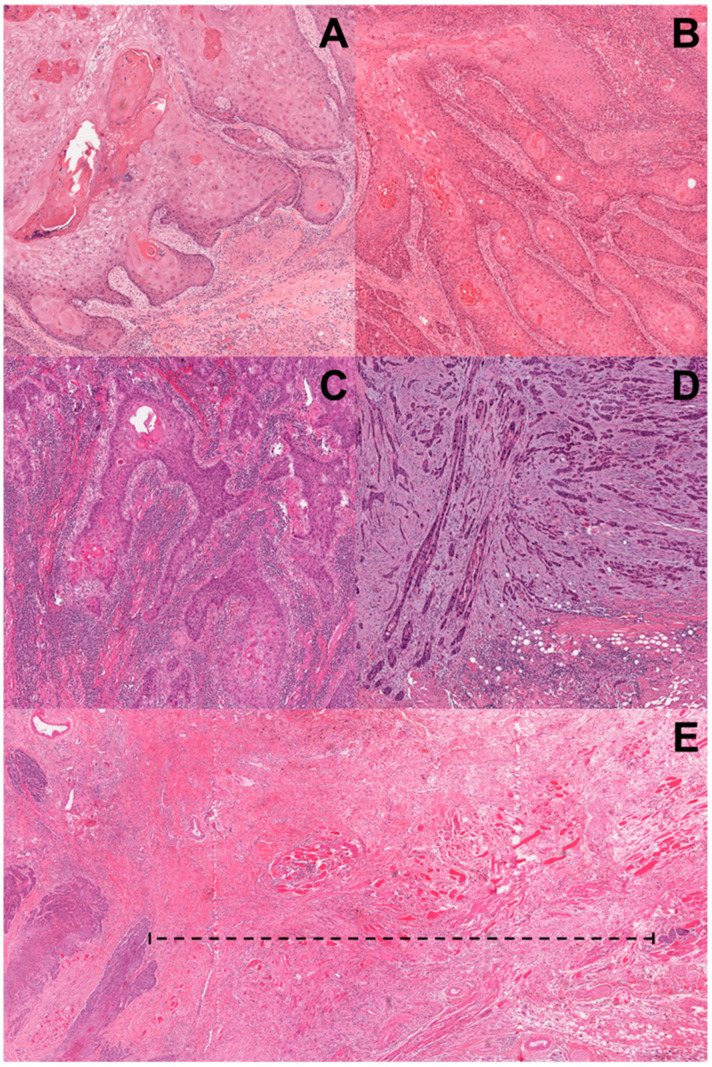
Histological representation of the Worst Pattern of Invasion in H&E-stained sections of OTSCC, according to Brandwein-Gensler M., et al. [13] (×20). (**A**) represents the POI-1 showing the presence of pushing and well-delineated infiltrating tumor borders. (**B**) represents the POI-2, in which tumor cells of the infiltrative front are assembled in solid cords, bands, and/or strands. (**C**) shows the POI-3: in this pattern, the infiltrative front consists of small groups or cords, each containing more than 15 infiltrating cells. (**D**) depicts the POI-4: the neoplastic cells at the infiltrative front are widespread in small groups of fever than 15 cells and/or in single cells. Finally, (**E**) represents the POI-5, defined as the presence of tumor satellites of any size that are ≥1 mm away from main tumor or next closest satellite with intervening normal tissue (not fibrosis) at the tumor/host interface. The dashed line indicates the minimum perpendicular distance between the deepest portion of the tumor and the satellite tumor.

Currently, the literature mainly refers to the risk score proposed by Brandwein-Gensler et al., which classified the well-delineated infiltrating tumor borders as “POI-1”; a neoplastic infiltration with solid cords, bands, and/or strands as “POI-2”; small groups or cords of infiltrating cells (*n* > 15) as “POI-3”; a widespread neoplastic dissociation in small groups (*n* < 15) and/or in single cancer cells as “POI-4”; and the presence of tumor satellites beyond 1 mm from the main tumor or next closest neoplastic satellite with intervening normal tissue (not fibrosis) at the tumor front as “POI-5”. The presence of small nests and/or single cancer cells and the extratumoral perineural and/or lymphovascular invasion with trapped nerve are considered tumor satellites (Figure 1). Furthermore, these authors introduced the Worst Pattern of Invasion (WPOI) as a new parameter considering the highest POI score, no matter how focal. WPOI scoring is independent of the extent, with no minimal cut-off value defined; accordingly, the presence of a single small tumor group (*n* < 15 cells) or even an isolated infiltrating tumor cell is considered positive [13].

In OSCC, the WPOI was reported as an independent prognostic factor for locoregional recurrences (LRRs), lymph node metastasis (LMN), overall survival (OS), disease-specific survival (DSS) [17,18,23,24], and infiltration of the cortical and medullary bone tissue [25,26], regardless of the oral subsite and the pathological stage. Several authors suggested the evaluation of the WPOI to lead the lymph node surgery and the postoperative management and to determine the occulted LNM in early OSCC [27,28,29]. To date, to simplify and improve OSCC prognostic stratification, the 8th edition of the AJCC staging system recommended to evaluate only the WPOI-5. However, it is still considered a minor prognostic parameter due to the lack of quantitative criteria, the heterogeneity of the infiltrative front, and the complex assessment of the tumor satellites.

Therefore, this comprehensive review summarizes the current knowledge of the WPOI with the aim of highlighting its future perspectives in oral cancer management.

## 2. Relevant Sections

### 2.1. Methodology

The study is a narrative review that extensively and critically analyzes the clinical and prognostic role of the Worst Pattern of Invasion in oral squamous cell carcinoma. The database search was conducted using the terms “(oral OR tongue OR lingual OR buccal OR gingiva OR mouth cancer OR squamous cell carcinoma OR tumour OR neoplasm) AND (prognosis OR predict OR survival OR recurrence OR mortality OR metastasis) AND (pattern of invasion OR worst pattern of invasion OR tumour invasion OR tumour pattern)” in the databases of PubMed, Scopus, and Web of Science. The selection process used a two-step screening technique. Step one involved screening the title and abstract of all studies identified during the literature search to determine which studies would proceed to full-text screening. In step two, the reviewers assessed each study’s eligibility by reading the full text. The inclusion criteria for study selection were as follows: (a) patients with OSCC, regardless of their pathological stage and site; (b) OSCC with all different grades of WPOI; (c) studies providing information or correlations between the WPOI and survival outcomes; (d) observational studies (i.e., case-control, cohort, and cross-sectional studies), retrospective studies, narrative reviews, systematic reviews, and meta-analyses; (e) articles published in the English language. Case reports, publications in languages other than English, conference abstracts, and animal sample studies were excluded from the narrative review. Moreover, all duplicates were removed.

### 2.2. Worst Pattern of Invasion Scores in Oral Squamous Cell Carcinoma: Historical Perspective and Current Trends

From the first observations regarding a possible relationship between the heterogeneity of tumor infiltration and cancer aggressiveness, different POI scores have been proposed in head and neck cancer. The initial point-based grading system of the mode of invasion was made by Jakobsson et al., which classified the “mode of invasion” of laryngeal carcinoma in four grades [21]. This grading system was subsequently modified by several authors and applied to other head-neck tumors. The “pattern of invasion” was firstly introduced by Crissman et al. to analyze the tumor cohesiveness and the association of the infiltrative neoplastic cells at the tumor–stroma interface. They demonstrated that the POI was the most significant histologic variable in predicting survival outcomes of OSCC patients [19]. At the same time, Yamamoto et al. modified the Jakobsson criteria, subdividing the Grade 4 into two categories: (i) Grade 4C, describing a cordlike type of invasion; and the (ii) Grade 4D, describing a widespread type of diffuse infiltration of single and/or small groups of neoplastic cells [22]. Later, Anneroth and Hansen proposed a new malignant grading system, recommending the evaluation of POI as a distinctive parameter compared to the other morphological features. The authors defined four POI categories: (i) Grade 1 corresponding to neoplasms with pushing, well-delineated borders; (ii) Grade 2 to neoplasms with infiltrating, solid cords, bands, and strands; (iii) Grade 3 to neoplasms with small groups of cells (>15 cells) or thin infiltration cords, regardless the number of cells; (iv) Grade 4 to neoplasms with a marked diffuse, widespread cellular invasion in single neoplastic cells or in small groups of cells (<15 cells) [30]. To date, several authors have been using the scoring system of Yamamoto et al. [15,31] and Anneroth and Hansen [17,18,32,33] for the WPOI assessment. However, the introduction of a fully developed WPOI scoring system by Brandwein-Gensler et al. represented a turning point in the study and classification of this parameter, and currently, it is the most widely used in the literature [23,24,25,29,31,32,34,35,36,37,38,39,40,41,42,43,44,45,46,47,48,49,50,51,52,53,54,55].

Most recently, this score was modified by Chang et al. to reduce its complexity and the overlap with the definition of tumor budding (TB), as both describe the loss of cellular cohesion and the active tumor invasion. The WPOI-4 definition seems to overlap with those of TB, which may result in a potential bias in evaluating the predictive value of both factors. Also, the cell counting in the WPOI-4 is time-consuming, subjective, and sensitive to variation caused by the slide section thickness. The modified WPOI (mWPOI) system does not consider the number of tumor cells per island, but it is focused to the tumor border and the distance from normal tissue. The mWPOI-1 is assigned to tumor nests confined within the main tumor boundary; mWPOI-2 refers to dispersed pattern types 1 and 3, ranging from minute protruding tumor focus to extensive tumor satellites but with <1 mm between tumor satellites; mWPOI-3 corresponds to an extensive dispersed pattern with tumor satellites with at least 1 mm of intervening normal tissue. Compared to the original classification, the mWPOI showed a lower LNM rate (5.4% vs. 10.0%) for the non-aggressive pattern and a higher sensitivity for LNM (94.3% vs. 85.7%) for the aggressive pattern. Moreover, it demonstrated a superior interobserver agreement than the original WPOI (k value = 0.98 vs. 0.53) [16].

Heerema et al. tried to verify the most reproducible WPOI scoring system; however, all of the 2-level categorized (WPOI-1/2 vs. WPOI-3/4: k = 0.400; WPOI-1/2 vs. WPOI-3: k = 0.455), the 4-level categorized (WPOI-1/5: k value = 0.346), and the 5-level categorized (WPOI-1/5: k value = 0.319) demonstrated a low interobserver agreement [32].

To date, only one study adopted a quantitative system to define the aggressive WPOI: (i) >50% of the tumor showing an aggressive WPOI; (ii) >20% of the tumor showing an aggressive WPOI; or (iii) any of the tumors, no matter how limited in extent, showing an aggressive WPOI. All the cut-offs displayed moderate interobserver agreement; in particular, the 50% and the 20% cut-offs demonstrated the higher agreement (K = 0.58, 95% CI 0.54–0.62), followed by the WPOI (К = 0.43, 95% CI 0.39–0.46). Certainly, the lack of quantitative criteria for the WPOI designation contributed to the conflicting results, as many tumors show heterogeneity in the infiltrative front with a combination of aggressive and non-aggressive patterns [14].

### 2.3. Worst Pattern of Invasion Risk Model in Oral Squamous Cell Carcinoma

The first WPOI risk model was the High-Risk Score (HRS) proposed by Brandwein-Gensler et al. The HRS is a three-tier system evaluating the WPOI, PNI, and the lymphocytic host response (LHR) to predict LRR and OS in OSCC [13] (Table 1). The HRS was demonstrated to predict the survival outcomes both in patients treated with surgery, with or without neoadjuvant therapy, and with surgery and postoperative radiation therapy (RT), suggesting that high-risk patients should benefit from adjuvant RT. These data were confirmed both in early- and advanced-OSCC [16,56].

Noteworthy, a clinical translation of the WPOI from a pathological assessment to high-resolution imaging evaluation has been recently demonstrated. A significant relationship between the HRS and the tumor front appearance based on a high-frequency intraoral ultrasound (IOUS) was observed: high-risk patients had a significant association with an irregular tumor front. Moreover, the IOUS demonstrated high accuracy values in predicting an HRS score ≥3 [34]. Another prognostic model has been proposed for the early OSCC risk stratification. By matching the POI and the tumor stage, Siriwardena et al. showed that high-risk patients could have metastases up to cervical nodal level 3, suggesting a selective neck dissection including levels I, II, and III as the minimum (Table 1) [18].

To identify LNM in early OSCCs, Shimizu et al. proposed to evaluate the WPOI, POI, and TB based on biopsies and surgical specimens by using pan-cytokeratin staining and to identify those areas with >5 buds and the POI-4C/4D patterns (Table 1). In the presence of these morphological features, a short-term clinical and radiographical follow-up (e.g., every one or two weeks for 6 months) should be planned. Moreover, in the case of clinical signs of LNM, a neck dissection should be conducted as soon as possible. However, with the presence of more than 10 buds and a POI-4D or WPOI-5 pattern in the same histological section, a wide surgical resection and an elective neck dissection (END) should be performed [15].

Also, De Silva et al. developed a model for predicting the LNM in OSCC, based on clinical (age, oral subsite) and histopathological (pT stage, POI, Depth of Invasion [DOI]) parameters (Table 1). They found a severe risk of nodal metastasis in pT3 and pT4 OSCC with POI-4, pT3 OTSCC with POI-4, and pT4 OTSCC with POI-3 or POI-4, regardless of the DOI (Table 2) [17].

Recently, the Histological Risk Model (HRM) has been proposed to predict the LNM and survival outcomes of early OTSCC, based on the mWPOI and TB (Table 2). Intermediate- and high-risk patients had a higher rate of LNM and LRR compared to the low-risk group, suggesting that an END should be recommended for these patients [16]. These data were also confirmed by Almangush et al. The high-risk group had a significantly lower DSS (HR: 2.54, 95% CI: 1.48–4.37, *p* < 0.001) compared to the low-risk group. These data suggested that high-risk patients require a multimodality treatment strategy with a close clinical follow-up [57]. However, no prognostic value of the HRM system was demonstrated in patients with OSCC at all clinical stages, and the WPOI and TB showed superior discrimination compared to the modified WPOI and modified TB [58].

### 2.4. Sample Detection of Worst Pattern of Invasion in Oral Squamous Cell Carcinoma

Most of the literature studies have evaluated the WPOI based on surgical specimens, while only four studies focused on the WPOI in incisional biopsies [24,49,59,60]. Several limitations hinder its accurate assessment in incisional biopsies, such as the small sample size, the lack of the infiltrative front, the sample fragmentations, artifacts, and extensive necrosis. However, some authors highlighted the potential usefulness of a preoperative evaluation of the WPOI in representative biopsies, including the deepest part of the primary tumor [24,60]. Only one author demonstrated the usefulness of the preoperative WPOI as an independent prognostic factor for the worst OS (WPOI-4 vs. WPOI-1: H.R. 3.3; 95% CI 1.1, 9.9, *p* = 0.04) [60]. On the contrary, other authors reported no significant correlation between the preoperative WPOI and survival outcomes in OSCC patients [24]. However, in early OSCC, it has been demonstrated that biopsy POI (BPOI)-4 perfectly predicts postoperative WPOI-4 and WPOI-5 and strongly predicts the DOI. Moreover, BPOI-1/2 patients showed a better OS, DFS, and recurrence-free survival and a lower DOI value compared to BPOI-4 cases, suggesting that BPOI-4 should be used as a sensitive parameter capable of identifying patients with biologically more aggressive tumors, for whom more radical surgical approaches should be considered compared to BPOI-1/2 patients [24].

Recently, the implementation of the WPOI in the machine learning models for early-OTSCC preoperative biopsies demonstrated a significant improvement in predicting occulted LNM compared to conventional statistical methods and single predictors [49].

Finally, in early OSCC, the WPOI-5 evaluation on intraoperative frozen sections significantly predicted the occulted LNM compared to WPOI-3/4. Moreover, it has been shown that WPOI-5 can be accurately identified intraoperatively (92.2%), suggesting that WPOI-5 OSCCs should undergo END. If WPOI-5 is identified only based on a permanent section, the patient can return to the operating room for more radical surgery [40]. The parameters and the modalities of WPOI detection are reported in Table 3.

### 2.5. Correlations Between Worst Pattern of Invasion and Histopathological Features in Oral Squamous Cell Carcinoma

Significant correlations were found between WPOI-5 and the nodal stage (*p* = 0.001), LVI (*p* = 0.006), PNI (*p* = 0.001), DOI > 4 mm (*p* = 0.001), and pT classification (*p* = 0.011) [13,36,37,43,44,48,61,62] and the budding number ≥ 5 (r = 0.438, *p* < 0.001) [29]. The WPOI 5 group also showed an increased DOI compared to the two WPOI ≤ 4 groups (14 vs. 4 mm, *p* < 0.01) [63].

Also, an aggressive pattern (WPOI-4/5) was significantly correlated with the PNI (*p* = 0.002) [42,64], DOI (*p* < 0.033), grade of differentiation (*p* < 0.002) [44], margin status (Pearson x^2^, *p* = 0.000) [13], nodal involvement (*p* = 0.018), and TB (*p* = 0.036) [62].

However, literature data are extremely variable; some authors did not show any significant association between the WPOI and tumor margin, bone involvement, lymphovascular invasion, tumor size and stage, and lymph node status [44,61,62,64]. Moreover, no association was observed between the WPOI and ENE; therefore, it should be considered independently in the treatment planning [61,62].

### 2.6. Prognostic Role of Worst Pattern of Invasion in Oral Squamous Cell Carcinoma

To the AJCC staging system, the WPOI represents an additional factor recommended for clinical care. It has been demonstrated as an independent prognostic factor of LRR [13,16,27,29,37,41,44,45,47,56,65,66], LNM [14,16,17,18,23,31,35,36,38,46,48,51,53,54,55,67,68,69,70] OS [13,23,36,37,39,43,45,50,54,65], DFS [15,24,29,41,44,65,71,72], and DSS [27,28,32,37,45,46,56,65], regardless of the oral subsite and the pathological stage (Table 4).

Aggressive WPOI tumors had the worst OS (HR = 2.2) with homogeneous pooled data (I^2^ = 0%) [65]. In particular, WPOI-4 and WPOI-5 tumors have 2.3-fold and 3.9-fold worse OS compared to WPOI-1/3 cases, respectively. The WPOI-5 was independently associated with OS both as satellite nodules (OR = 6.6) and extratumoral LVI (OR = 10) [45].

Moreover, the mortality rate of WPOI-5 patients is equal to 70% compared with 26% for WPOI-1/4 patients [41,50,65].Therefore, an aggressive WPOI represents an independent predictor of OS, and the WPOI reaches the highest HR (1.64) compared to the other predictors [24,36,39,43,50,73]. However, some authors did not confirm the prognostic role of WPOI-5 in OS (OSCC: HR 1.36; 95% CI; *p* = 0.5; OTSCC: HR 2.58; 95% CI; *p* = 0.09) [59,63,74].

Aggressive WPOI patients have a 3.37-fold higher risk of LRR compared to WPOI 1-3 OSCCs. Furthermore, these patients showed a 4.67-fold and 1.58-fold higher risk of DDS and DFS than WPOI-1/3 cases, respectively. However, high heterogeneity pooled data were detected (Table 3). This heterogeneity could be attributed to the differences in the patient population, study design, and methodologies. In fact, some studies did not consider potential confounding factors, such as patient demographics, tumor stages, and treatment modalities, which could influence the relationship between the WPOI and survival outcomes. For example, the only inclusion of early OSCCs might lead to the under-representation of WPOI-5 patients. Moreover, the various WPOI definitions and categorization cut-offs could have significantly influenced the results [65,75].

Many studies demonstrated a significant correlation between the WPOI and LNM [17,18,33,36,38,44,48,67,70,76,77,78], while only two studies did not confirm the role of the WPOI in predicting LNM [14,35]. These discrepancies could be attributed to the small sample size and to the different methodologies. Both the studies retrospectively evaluated patients with different pathological stages and tumor natures, undergoing different treatment modalities. Beggan et al. only included floor of the mouth OSCC, while, in the study of Deshapande et al., the POI-4 and the POI-5 were considered as the WPOI. Finally, an aggressive WPOI was also significantly associated with bone infiltration compared to WPOI-1/3 OSCCs [14,35].

Noteworthy, WPOI-5 represents the most significant factor for predicting occulted LNM in early OSCCs, demonstrating a LNM rate equal to 62.5%, with respect to 34.8% for WPOI-1/4 cases [23,39,40,50,51,52,53,68,75,78]. In addition, WPOI-5 tumors have a 2.6-fold higher risk of LRR, a 6.3-fold worse DSS, and a 6.4-fold higher risk of occulted LNM compared to WPOI-1/4 [53,78]. Similarly, aggressive tumors (WPOI 4/5) have an LNM rate equal to 74.3% compared to 25.7% for non-aggressive ones (WPOI 1/3) [51].

In early OTSCC, a predictive nomogram demonstrated that WPOI-5 most contributed to the survival prediction: WPOI-5 tumors showed a 14.9-fold higher risk of LRR and a 6.9-fold worse DSS compared to tumors without WPOI-5 [79].

**Table 4:** Systematic review and meta-analysis regarding the prognostic role of Worst Pattern of Invasion in oral squamous cell carcinoma.

Authors (Year)	Results	HR (95% CI)	I^2^
Binmadi et al. (2023) [65]	WPOI-4 and WPOI-5 are significant associated with worst OS, DFS, DSS, LRR, and LRFS in OSCC.	OS: 2.17 (1.79–2.55) DSS: 4.67 (1.30–8.04) DFS: 1.58 (1.10–2.07) LRR: 3.37 (2.62–4.12) LRFS: 1.68 (1.03–2.33) Mortality rate: 3.86 (2.84–4.88)	0.0% 93.47% 68.6% 8.8% 46.52% N.A.
Elseragy et al. (2022) [75]	WPOI is significantly associated with worst DFS in OTSCC.	DFS: 1.95 (1.04–3.64)	28%

WPOI: Worst Pattern of Invasion; OS: overall survival; DFS: disease-free survival; DSS: disease-specific survival; LRR: loco regional recurrence; LRFS: local recurrence-free survival; OSCC: oral squamous cell carcinoma; OTSCC: oral tongue squamous cell carcinoma; N.A.: Data not available.

## 3. Discussion

### Clinical Implications and Therapeutic Impact of Worst Pattern of Invasion in Oral Squamous Cell Carcinoma

According to the National Comprehensive Cancer Network (NCCN), early-stage OSCCs with a DOI ≥ 4 mm should be treated with surgical resection and ipsilateral neck dissection. An END can be performed also in tumors with a DOI equal to 2–4 mm, according to the clinical judgment. However, performing an END only based on the DOI could result in overtreatment, because LMNs were detected in 5.6% and 16.9% of tumors with a DOI equal to 3 mm and 4 mm, respectively [80]. Moreover, a bilateral neck dissection and a postoperative RT, or adjuvant systemic therapy, are considered based on the primary tumor site and on adverse risk factors, respectively. In advanced tumors, the surgical resection and an ipsilateral or bilateral neck dissection, and an adjuvant systemic therapy, are performed depending on the risk factors. Among the adverse risk factors, the WPOI resulted in a strong prognosticator of LNM, such that it could be relevant to select patients undergoing END and/or adjuvant radiation or systemic therapy (Table 5). Since the WPOI is a histopathological feature of the primary tumor that reflects the pattern of the neoplastic cell arrangement at the invasive front, it represents the area of active tumor invasion at the tumor–host interface. Consequently, “high-grade” WPOI tumors may exhibit an enhanced ability to infiltrate adjacent tissues, including lymphatic, vascular, perineural, osseous, muscular, and glandular/adipose structures. In this regard, OSCC can quickly infiltrate the lymphatic vessels and spread to loco-regional lymph nodes. It occurs through the dissemination of emboli from the primary tumor to regional lymph nodes, through internodal lymphatic vessels or through direct extracapsular spread. Furthermore, the AJCC had recognized the presence of LNM as the most valuable prognostic factor of the OSCC outcome and a strong indicator of the OSCC ability to metastasize, resulting in a 50% reduction in patient survival. Therefore, the WPOI could be considered an indirect marker of LNM rather than a direct prognostic factor for survival, as OS and DFS are influenced by multiple variables beyond the disease stage, including treatment modalities and patient-specific factors.

In early OTSCCs, the immunostaining of frozen sections has been recommend to identify high-risk tumors to submit them to END during the surgical treatment of the primary tumor [27]. In early OSCCs, the evaluation of the POI and the TB based on biopsies and surgical specimens using immunostaining to identify the occulted LNM has been suggested. Tumors with more than five buds and a POI-4C/4D pattern should undergo a short-term follow-up (every one or two weeks for 6 months) with or without imaging, and a neck dissection should be performed, as soon as possible, in the presence of any clinical signs of nodal relapse. Instead, in the simultaneous presence of more than 10 buds and POI-4D or WPOI-5 patterns in the same section, a wide resection and END should be conducted [15].

Kohler et al. found that the extent of the surgical margins depends on the WPOI: in patients with a non-aggressive WPOI, a cutoff of 1.8 mm was considered as ideal clear surgical margins, while in an aggressive WPOI, the ideal distance to the margins was 8.0 mm. Based on these data, the authors suggest that the WPOI can be used as a parameter to guide the extent of surgical margins, and it may impact the decision to perform adjuvant treatment in patients with OSCC [42]. Brandwein-Gensler et al. recommend adjuvant RT for high-risk, pT1-2, pN0-1 OSCCs, demonstrating a local disease-free benefit from adjuvant RT for these patients. On the contrary, no local disease-free benefit in administering adjuvant RT in low-risk and intermediate-risk patients was observed [13]. Other authors recommended documenting both WPOI-4 and WPOI-5 to detect high-risk OSCC to avoid any bias due to the low WPOI-5 rate and the overlapping of the WPOI-4 and TB definition [52]. These patients should be considered for radical surgery when compared with patients with WPOI-1/3. In particular, Pu et al. proposed the evaluation of BPOI-4 in a preoperative biopsy since it was demonstrated to effectively predict WPOI-4/5 in the postoperative samples [24,39]. Also, Yamauchi et al. suggested that a policy of no treatment until LNM appears is inappropriate for early-stage OSCC with WPOI-4/5. They recommended performing a neck dissection as early as possible after transoral resection or a pull-through resection and prophylactic neck dissection if an aggressive WPOI is detected in the preoperative sample [46].

Despite the clinical relevance of the WPOI and TB and the efforts to standardize and simplify histopathological systems being commendable for reducing interobserver variability and minimizing overlapping criteria, the findings of the current studies highlight some limitations. In particular, the mWPOI system was recently introduced to reduce the complexity of WPOI scoring and the overlap with the definition of TB, as both describe the loss of cellular cohesion and active tumor invasion. The WPOI-4 definition seems to overlap with those of TB, which may result in a potential bias in evaluating the predictive value of both factors. The mWPOI system does not consider the number of tumor cells per island, but it is focused on the tumor border and the distance from normal tissue. However, literature data are scarce and conflicting. The prognostic performance of the mWPOI system was confirmed in early-stage OTSCC [57], while no prognostic value was demonstrated in patients with OSCC at all clinical stages. Furthermore, the WPOI and TB showed superior discrimination compared to the modified WPOI and modified TB [79]. The significant prognostic differences between WPOI-3 and WPOI-4 emphasize the importance of maintaining these two categories. Moreover, incorporating single cells into the TB score does not appear to significantly enhance prognostic stratification. Furthermore, the restrictive definition of high-risk cases, which requires the presence of a tumor satellite, is relatively uncommon in OSCC [79].

The WPOI assessment was almost completely limited to surgical resection specimens, which may restrict its potential usefulness in preoperative risk stratification. Only one study demonstrated that preoperative BPOI-4 perfectly predicts postoperative WPOI-4 and WPOI-5 and BPOI-4 strongly predicts the DOI, suggesting that these patients should be considered for radical surgery. On the contrary, other authors reported no significant correlation between the preoperative WPOI and survival outcomes in OSCCs. The small sample size, the fragmentations, artifacts, and the necrosis, as well as the usual lack of an infiltrative front represented the main limitations of the WPOI evaluation based on incisional biopsies. Furthermore, incisional biopsies are not suitable to assess the highest WPOI and the tumor satellites.

Overall, the results of the studies regarding the prognostic value of the WPOI in OSCC should be taken carefully, due to the lack of a standardized detection method, a standardized scoring system, and a quantitative criteria defining the POI. Moreover, no minimal cut-off value has been established for the WPOI definition.

Several methodological inconsistencies are reported, in particular (i) the inter-observer agreement in the POI and WPOI assessment is hindered by the heterogeneity of the infiltrative tumor front and by the complex assessment of the tumor satellites; (ii) most of the studies are single-centre retrospective studies; and (iii) the most reliable WPOI value and scoring system is not established.

Despite these limitations, several studies demonstrated that the “aggressive” WPOI is significantly correlated with LNM in OSCC, suggesting an evaluation of the WPOI in pathological daily practice. Additional multicenter and prospective studies and accuracy analysis are required to standardize WPOI assessment methods and to establish the most accurate WPOI score and cut-off.

## 4. Conclusions and Future Directions

**Figure 2:**
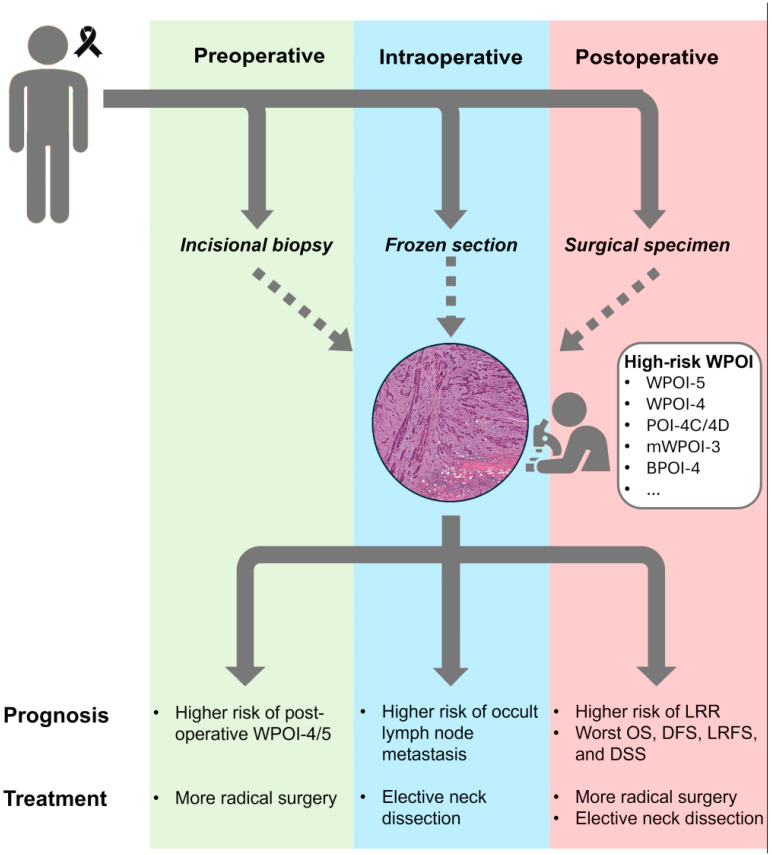
Schematic representation of the potential prognostic and therapeutic role of WPOI in routine pathological assessment.

The WPOI has been validated as a survival prognosticator of oral cancer patients, especially for early-stage tumors. Five WPOI patterns have been recognized; however, the College American on Pathology and the AJCC system guidelines supported only the distinction between WPOI-5 and other patterns, since the WPOI can be redundant in the reporting of the extratumoral PNI and LVI. However, it has been demonstrated that high-risk patients could benefit from wide resection of the primary tumor, an END, and an adjuvant RT to the primary site, despite the negative resection margins. Because of the clinical and prognostic relevance of the WPOI, it could simplify the stratification of the “individual risk” and the streamlining of “personalized treatment”. Therefore, its evaluation in pathological daily practice could offer the clinician a further parameter leading to multidisciplinary disease management (Figure 2). Additional multicenter and prospective studies and an accuracy analysis are required to standardize WPOI assessment methods and establish the most accurate WPOI score and cut-off.

Nowadays, the development of deep-learning-based methods in pathology has rapidly evolved over recent years, representing a promising tool for the automated evaluation of histopathological parameters, particularly those that are time-consuming to assess using conventional microscopy. In this context, the WPOI is of particular interest, together with other parameters such as TB and the tumor–stroma ratio (TSR), all of which originate from the need to identify features that act as hallmarks of epithelial–mesenchymal transition, capturing the heterogeneity of the TME and the complexity of the interactions between tumor cells and the surrounding stroma.

Some parameters are morphologically well-defined, such as TB, which is characterized by the presence of small clusters of tumor cells detaching from the invasive front, making it particularly suitable to automated evaluations using deep-learning-based algorithms [3]. Indeed, several studies have demonstrated the usefulness of AI-based systems in evaluating TB, highlighting their strong performance, with greater reliability, accuracy, and speed in tumor bud quantification compared with human operators [81,82]. On the other hand, TSR represents a more global metric, as it provides a comprehensive and integrated assessment of the overall tissue composition, requiring different deep-learning-based approaches for its automatic quantification. Despite some methodological challenges [83], several studies have recently highlighted the feasibility of using AI-based systems to evaluate TSR [84,85].

In this context, the WPOI shares morphological and conceptual features with both TB and TSR and is therefore affected by similar methodological challenges while also offering comparable opportunities for automation using AI-based approaches. Accordingly, the development and implementation of AI-based methods for the automated assessment of the WPOI will require dedicated algorithms trained and validated based on large, well-balanced OSCC cohorts, in order to address known limitations such as overfitting, class imbalance, and related sources of bias [86].

## Figures and Tables

**Table 1:** Worst Pattern of Invasion risk models in oral squamous cell carcinoma.

Author, Year	Parameters	Score	Comments
Brandwein-Gensler et al., 2005 [13]	PNI LHR WPOI	**0**: None PNI; Continuous band of LHR; WPOI 1-2-3. **1–2**: Small nerves; Large patches of LHR; WPOI-4. **3–7**: Large nerves; Little or none LHR; WPOI-5.	- The model is predictive in all therapeutic categories (neoadjuvant therapy and surgery, surgery alone, surgery, and adjuvant RT).
Shimizu et al., 2018 [15]	WPOI ITBCC	**Low-risk**: WPOI-1/4 and TB ≤ 4. **Intermediate-risk**: POI-4C/4D and TB 5–9. **High-risk**: WPOI-5; POI-4C/4D and TB ≥ 10.	- The model shows strong correlation between mode of invasion and TB. - TB is the most powerful prognostic marker of DFS.
De Silva et al., 2018 [17]	pT DOI POI	**Low-risk**: pT1 and POI-2/3/4; pT2 and POI-2/3. **Minor-risk**: pT2, DOI < 4 mm, and POI-4; pT3 and POI-2; pT4, DOI < 4 mm, and POI-2. **Moderate-risk**: pT3 and POI-3; pT4, DOI < 4 mm, and POI-3; pT4, DOI ≥ 4 mm, and POI-2. **High-risk**: pT3, DOI < 4 mm, and POI-4; pT4, DOI ≥ 4 mm and POI-4. **Severe-risk**: pT3, DOI ≥ 4 mm, and POI-4; pT4 and POI-4.	- The model provides an accurate stratification of patient’s risk, based on the pT parameters of the AJCC system.
Siriwardena et al., 2018 [18]	Stage POI	**Level 1**: POI-1 and S-3; POI-2 and S-1/2. **Level 2**: POI-1 and S-4; POI-2 and S-3; POI-3 and S-1/2. **Level 3**: POI-2 and S-4; POI-3 and S-3; POI-4 and S-1/2. **Level 4**: POI-3 and S-4; POI-4 and S-3. **Level 5**: POI-4 and S-4.	The model provides a clinical guide for the lymph node treatment in early and advanced OSCC. Early OSCC: - POI-2: I level dissection. - POI-3: dissection up to the II level. - POI-4: dissection up to III level. In S3 the dissection follows the grade of POI. Advanced OSCC - POI-1: dissection up to II level. - POI-2: dissection up to the III level. - POI-3: dissection up to IV level. - POI-4: dissection up to V level.

PNI: perineural invasion; DOI: depth of invasion; WPOI: Worst Pattern of Invasion; mWPOI: modified Worst Pattern of Invasion; LHR: lymphocytic host response; POI: pattern of invasion; TB: tumor budding; S: stage; RT: radiation therapy; DFS: disease-free survival; AJCC: American Joint Committee on Cancer.

**Table 2:** Worst Pattern of Invasion risk models in oral tongue squamous cell carcinoma.

Author, Year	Parameters	Score	Comments
De Silva et al., 2018 [17]	pT DOI POI	**Low-risk**: pT1 and POI-2/3; pT2, DOI < 4 mm, and POI-2. **Minor-risk**: pT1 and POI-4; pT2, DOI < 4 mm, and POI-3; pT2, DOI ≥ 4 mm, and POI-2. **Moderate-risk**: pT2, DOI ≥ 4 mm, and POI-3; pT3, DOI < 4 mm, and POI-2. **High-risk**: pT2 and POI-4; pT3, DOI < 4 mm, and POI-3; pT3, DOI ≥ 4 mm, and POI-2. **Severe-risk**: pT3/4 and POI-4; pT4 and POI-3; pT3, DOI ≥ 4 mm, and POI-3; pT4, DOI ≥ 4 mm, and POI-2.	- The model provides an accurate stratification of patient’s risk, based on pT parameters of the AJCC system.
Chang et al., 2024 [16]	TB mWPOI	**0–1**: mWPOI-1 and TB0/1; mWPOI-2 and TB0. **2–3:** mWPOI-1/2 and TB0/1/2; mWPOI-3 and TB0/1. **4**: mWPOI-3 and TB2	- mWPOI shows higher sensitivity for LNM and higher interobserver agreement respect to WPOI.

DOI: depth of invasion; mWPOI: modified Worst Pattern of Invasion; WPOI: Worst Pattern of Invasion POI: pattern of invasion; TB: tumor budding; LNM: lymph node metastasis.

**Table 3:** Parameters and modalities of WPOI detection in oral squamous cell carcinoma.

Parameters	Modalities Reported in Literature
Evaluation approaches	- Manual detection - Digital detection - Ultrasonography detection
Cut-off	- WPOI-5, WPOI-1/5, WPOI-1/4, WPOI-1/4C+4D, mWPOI, BPOI, UFOI - Dichotomic cut-off (positive/negative); (crumb/trabes); (cohesive/invasive) - Quantitative cut-off
Specimen	- Biopsy specimen - Frozen section - Surgical specimen
Oral subsite	- Oral - Tongue - Gingival–buccal complex - Mouth Floor - Buccal Mucosa

WPOI: Worst Pattern of Invasion; mWPOI: modified Worst Pattern of Invasion; BPOI: biopsy pattern of invasion; UFOI: ultrasonographic front of infiltration.

**Table 5:** Framework of lymph node management based on adverse risk factors.

Author	Parameters	Clinical Decision Making
AJCC 8th Ed. [5]	DOI	Elective neck dissection in tumors with DOI ≥ 4 mm
Shimizu et al. [15]	POI TB	Short-term follow-up and imaging in tumors with >5 buds and POI-4C/4D patterns. Wide surgical resection with elective neck dissection in tumors with >10 buds and POI-4D or WPOI-5 patterns.
Siriwardena et al. [18]	POI Stage	Early OSCC: - POI-2: dissection of I level. - POI-3: dissection up to the II level. - POI-4: dissection up to III level. In S3 the dissection follows the grade of POI. Advanced OSCC - POI-1: dissection up to II level. - POI-2: dissection up to the III level. - POI-3: dissection up to IV level. - POI-4: dissection up to V level.
Brandwein-Gensler et al. [13]	Stage	Surgery and adjuvant radiation therapy in pT1-2, pN0-1 OSCC.
Yamauchi et al. [46]	Stage WPOI-5	Elective neck dissection in early-OSCCs with WPOI-5 or WPOI-4.
Kohler et al. [42]	Surgical margin WPOI	Wide resection with surgical margins > 8 mm and adjuvant treatment in tumors with WPOI-4/5.

WPOI: Worst Pattern of Invasion; OSCC: oral squamous cell carcinoma; POI: pattern of invasion; DOI: depth of invasion; S: stage; AJCC: American Joint Committee on Cancer.

## Data Availability

No new data were created or analyzed in this study.

## Abbreviations

The following abbreviations are used in this manuscript:

AJCC	American Joint Committee on Cancer
BPOI	Biopsy Pattern of Invasion
DFS	Disease-Free Survival
DOI	Depth of Invasion
DSS	Disease-Specific Survival
END	Elective Neck Dissection
HRM	Histological Risk Model
HRS	High Risk Score
ITBCC	International Tumor Budding Consensus Conference
LNM	Lymph Node Metastases
LHR	Lymphocytic Host Response
LRR	Locoregional Recurrence
LVI	Lymphovascular invasion
mWPOI	Modified Worst Pattern of Invasion
NCCN	National Comprehensive Cancer Network
OS	Overall Survival
OSCC	Oral Squamous Cell Carcinoma
OTSCC	Oral Tongue Squamous Cell Carcinoma
POI	Pattern of Invasion
PPOI	Predominant Pattern of Invasion
PNI	Perineural Invasion
RT	Radiation Therapy
TB	Tumor Budding
TME	Tumor Microenvironment
WPOI	Worst Pattern of Invasion
